# Organized breast screening improves reattendance compared to physician referral: a case control study

**DOI:** 10.1186/s12885-015-1346-2

**Published:** 2015-04-26

**Authors:** Ilia Makedonov, Sumei Gu, Lawrence F Paszat, on behalf of the investigators of the Ontario Cancer Screening Research Network

**Affiliations:** 1 University of Toronto, Toronto, ON Canada; 2 Institute for Clinical Evaluative Sciences, Toronto, ON Canada; 3 Sunnybrook Health Sciences Centre, Toronto, ON Canada

**Keywords:** Breast cancer screening, Case control study, Reminder letters, Canada, Ontario Breast Screening Program

## Abstract

**Background:**

The Ontario Breast Screening Program (OBSP) is a population-based breast screening programme, not requiring physician referral. OBSP invites women by mail to book their next screens. However, women who do not participate in the OBSP, may be referred by physicians to non-OBSP mammography facilities, which do not remind women to book their next screen.

**Methods:**

We identified women without breast cancer prior to June 30, 2011, having bilateral mammography (M) during a baseline period at age 50 – 69 at OBSP or non-OBSP facilities, and during a re-exposure period, at the same facility type. We used a case-control design to study the association of facility type and having M during an outcome period. Cases were women failing to receive the outcome M. Controls were matched by age, census tract, and socioeconomic status. Exposure was baseline facility type. Covariates were comorbidity, residential mobility, and primary care physician (PCP) characteristics. Conditional logistic regression analysis was performed.

**Results:**

Cases were less likely to have been screened at OBSP facilities. Failure to receive the outcome M was associated with having moved after re-exposure M (OR = 1.61, 95% confidence interval (CI) 1.52, 1.71), having a male PCP (OR = 1.05, 95% CI 1.02, 1.05), or a higher Charlson score (OR = 1.06 per unit increase, 95% CI 1.03, 1.09). Having re-exposure M at an OBSP facility (OR = 0.18, 95% CI 0.18, 0.19)., having a Canadian trained PCP (OR = 0.83, 95% CI 0.8, 0.87), and having a PCP one year after the re-exposure M (OR = 0.81, 95% CI 0.68, 0.97) were protective against failure to receive the outcome M.

**Conclusions:**

The OBSP, not requiring physician referral, and inviting women by mail to book their next screen, is associated with a lower probability of failure to reattend for subsequent screening than screening by PCP referral to non-OBSP facilities.

## Background

Several randomized trials and meta analyses have shown that periodic screening with mammography reduces breast cancer mortality among women by around 14 to 35 percent with regular screening [1-3], depending on age and the specific trial [4,5]. Current Canadian guidelines recommend screening mammography every 2-3 years for average risk women aged 50-69 [6].

### Breast screening in Ontario

The Ontario Breast Screening Program (OBSP) is a population-based breast screening program for women 50 – 69 years of age previously unaffected by breast cancer, and does not require women to be referred by a physician. The OBSP invites women by mail to book their next screen, when it comes due. The OBSP has a website with information for physicians and for screen-eligible women. It does not conduct risk assessments or counsel women at entry or at reattendance. It sends screening results to the woman, and to a primary care physician if she has identified a physician to the programme.

Women who are eligible for breast screening, but who do not attend the OBSP for whatever reason, may be referred by physicians to non-OBSP mammography facilities for screening mammography (M). These facilities do not accept self-referral by age-eligible women (women may not book their own appointments) and do not remind the woman or the physcian when the next screen is due. For some women a non-OBSP facility may be more convenient. These facilities send the results of each screen to the referring physician, but not to the woman.

Previous work from the OBSP has identified several factors that affect reattendance for the next screen [7], but this work did not compare OBSP reattendance with reattendance at other facilities. The effect of patient reminders on mammography attendance has previously been studied in other jurisdictions [8-10]. In a randomized trial, Mayer et al. found that the group receiving a reminder letter from the mammography facility had a 46.6% return rate and the group receiving no letter had a 28.3% return rate [8].

### Primary care in Ontario

In Ontario, primary care is provided chiefly by non-specialist physicians, who may or may not have had 2 or 3 years post graduate training in family medicine.

### Universal single governmental health insurance in Ontario

All women who are permanent residents or refugees in Ontario are ensured by the government for bilateral mammography at both types of mammography facility. The Ministry of Health provides lump sum financial incentive payments to physicians who report various levels of participation among their eligible patients. These incentives apply to both OBSP and non-OBSP screened women. However, we have recently shown that these incentives do not increase attendance for screening mammography in Ontario [11].

### Ethics

The Sunnybrook Health Sciences Center Research Ethics Board approved this study (project number PO940 010). Written consent was not obtained as the study was entirely registry-based and did not involve biological samples or contact with participants. All patient and physician data were anonymized.

## Methods

### Data sources

The Registered Persons Database (RPDB) is a roster of beneficiaries of the Ontario Health Insurance Plan (OHIP), for which all permanent residents and refugees are eligible free of charge. The RPDB contains the postal code of each beneficiary, allowing the linkage of each beneficiary to Statistics Canada data on residence and census-level variables, such as socio-economic status. From the OHIP physician claims billing database we obtained records of mammography, breast ultrasound, breast MRI, breast biopsies and breast surgery from 2005 - 2011. All records are identified by the service code, date of service, physician identifier and an encryption of the unique numeric health insurance number which allows deterministic linkage of each beneficiary to all provincial databases. The physicians billing for services to each beneficiary are analyzed, in order to determine if each beneficiary has, or does not have, a usual primary care physician. From the OBSP database we obtained all dates of breast screening between 2005 and 2011. From the Ontario Cancer Registry we identified all dates of diagnosis of breast cancer prior to December 31, 2011. Information about physician age, sex, country of education, and years in practice was obtained from the Corporate Providers’ Database (CPDB).

### Study population

We identified women without breast cancer or mastectomy prior to June 30, 2011, having bilateral mammography (M) during a baseline period at ages 50 – 69 at OBSP or non-OBSP facilities, and during a re-exposure period at the same type of mammography as at baseline. Women were excluded if baseline M or re-exposure M were followed by other diagnostic imaging of the breast, breast biopsy or breast surgery, or, if no usual primary care physician could be identified as of the date of re-exposure M.

Women who received the outcome M within the expected interval of 11 months to 30 months following re-exposure M comprised the controls, while those who failed to receive the outcome M comprised the cases. Multiple controls were matched to cases by age, census tract, and urban median household income quintile/rural residence at January 1, 2008.

### Exposure to breast screening and definition of baseline, re-exposure and outcome periods

The baseline period was December 1, 2005 to June 30, 2007. The re-exposure period was January 1, 2008 to June 30, 2008, and the interval between baseline M and re-exposure M was defined as 11 to 30 months. This provides a population of observations for whom the pattern of periodic screening is established in the baseline and re-exposure periods. If the baseline M and re-exposure M had been provided by the OBSP, an invitation letter would have been sent to each woman, approximately 24 months later. The outcome period was defined as 11 to 30 months after the re-exposure M, and ended no later than December 31, 2010.

### Study design

We used a case-control design to study the association between the type of mammography facility, and the failure to receive the outcome M. Cases were women who failed to receive the outcome M. Controls did receive the outcome M and were matched to controls by age, census tract, and socioeconomic status. Exposure was the type of mammography facility for each women. Covariates were comorbidity, residential mobility, and primary care physician (PCP) characteristics.

### Definition of adjusting variables

#### Cumulative (5 year) Charlson score as of January 1, 2008

Using the Deyo modification of the Charlson score for administrative data, we calculated the Charlson score accumulated during the 5 years prior to the re-exposure date for all women.

#### Having a primary care physician (yes/no) one year after re-exposure M.

Women had an identifiable primary care physician on the date of the re-exposure M, as an eligibility criterion, ascertained from OHIP records. We ascertained whether or not each woman had a usual primary care physician (PCP) twelve months after the re-exposure M. Any woman might not have a usual PCP at that time, because of personal preference, retirement or relocation of the PCP, or the woman having moved to a different community. OHIP does not assign any beneficiary to a particular physician, it is the responsibility of the beneficiary to find one who will accept her as a patient.

#### Gender of primary care physician

For each woman, the gender of her primary care physician was extracted from the CPDB.

#### Location of medical education of primary care physician

The CPDB contains name of the medical school attended by each physician, and the name of the country in which the medical school was located. We categorized the physician of each woman as Canadian-trained, Anglosphere – trained (US, UK, Ireland, Australia, New Zealand, South Africa), or Other-trained.

#### Years in practice of primary care physician

The number of years spent in practice were extracted for each PCP from the CPDB.

#### Postal code change one year after 2008 mammography

We identified for each woman whether she had moved by 12 months following the index date by searching for a change in postal code. This would identify a potential barrier to her receiving an invitation letter for reattendance at breast screening thereafter due to delays in recording address changes, and might signify that the woman would no longer be able to attend her usual PCP.

### Statistical analyses

Conditional logistic regression was conducted to estimate the odds ratio for failure to receive the outcome M, treating the type of mammography facility of the baseline and re-exposure M as the exposure variable, adjusting for the variables described above.

## Results

The study population comprised 105,665 women, whose description is tabulated in Table 1. There were more women in the upper income quintiles compared to lower. On the index date, 93.6% of women had a Charlson comorbidity score of ‘zero’. 60.6% of the women had a male primary care physician and 77% of the women had a Canadian-educated primary care physician. We were able to identify a primary care physician for 99.2% of the study population one year after the date of the re-exposure M. At that time, 5.9% of women had moved to a different address (evidenced by a change in postal code).

**Table 1:** **Characteristics of the study population**

Factor		All women (n = 105,665)	
	Entire sample (% of total)	Cases (% of total)	Controls (% of total)
Socioeconomic status quintiles			
First urban quintile (lowest)	12,499 (11.8%)	3,612 (13.4%)	8,887 (11.3%)
Second urban quintile	15,686 (14.8%)	4,364 (16.2%)	11,322 (14.4%)
Third urban quintile	16,523 (15.6%)	4,432 (16.4%)	12,091 (15.4%)
Fourth urban quintile	19,040 (18.0%)	5,011 (18.6%)	14,029 (17.8%)
Fifth urban quintile (highest)	23,555 (22.3%)	5,752 (21.3%)	17,803 (22.7%)
Rural	18,259 (17.3%)	3,794 (14.1%)	14,465 (18.4%)
Missing	103 (0.1%)	35	68
Age at initial scan			
50-54	28, 090 (26.6%)	7,710 (28.6%)	20,380 (25.9%)
55-59	32,413 (30.7%)	8,001 (29.6%)	24,412 (31.1%)
60-64	26,663 (25.2%)	6,501 (24.1%)	20,162 (25.7%)
65-69	18,499 (17.5%)	4,788 (17.7%)	13,711 (17.4%)
Cumulative Charlson score on index date			
0	98,885 (93.6%)	25,135 (93.1%)	73,750 (93.8%)
1	3,762 (3.6%)	1,049 (3.9%)	2,713 (3.5%)
2	2,115 (2.0%)	548 (2.0%)	1,567 (2.0%)
≥3	903 (0.9%)	268 (1.0%)	635 (0.7%)
Women by gender of their PCP at index date			
F	41,616 (39.4%)	10588 (39.2%)	31028 (39.5%)
M	64,049 (60.6%)	16412 (60.8%)	47637 (60.5%)
Women by country of education of physician on index date			
Canadian	82,020 (77.6%)	20322 (75.3%)	61,698 (78.5%)
Anglosphere	7.639 (7.2%)	1995 (7.4%)	5644 (7.2%)
Other	16,006 (15.1%)	4683 (17.3%)	11323 (14.3%)
Women by years since graduation of physician at index date			
<10	5,573 (5.3%)	1,352 (5.0%)	4,221 (5.4%)
10-19	21,621 (20.5%)	5525 (20.5%)	16,186 (20.6%)
20-29	37,053 (35.1%)	9,522 (35.3%)	27,531 (35.0%)
30-39	30,671 (29.0%)	7,758 (28.7%)	22,913 (29.2%)
> = 40	10,657 (10.1%)	2,843 (10.5%)	7,814 (9.8%)
Women who still have a PCP at year one after index date			
Yes	104,801 (99.2%)	26801 (99.3%)	78000 (99.2%)
No	854 (0.8%)	199 (0.7%)	655 (0.8%)
Women whose Postal code has changed from index date to year one			
Yes	6,264 (5.9%)	2152 (8.0%)	4112 (5.1%)
No	99,401 (94.1%)	24848 (92.0%)	74553 (94.9%)
Baseline and re-exposure mammography facility			
OBSP	63,264 (59.9%)	8,964 (33.2%)	54,230 (69.0%)
Non-OBSP	42,401 (40.1%)	18,036 (66.8%)	24,365 (31.0%)

Women in the lowest urban income quintile were less likely to reattend for screening after the index date (71.1%) compared to the highest income quintile. (75.6%). Women with a Charlson comorbidity score = 3, on the index date, were more likely to fail to receive the outcome M (only 70.3% reattended) compared to those with a score of ‘zero’ (74.6% reattended).

Among the study population, 59.6% had received their baseline and re-exposure M at an OBSP facility, and 40.4% elsewhere. Among women with a Canadian-educated PCP, 24.8% failed to receive the outcome M, compared to 26.1% of those with a PCP educated elsewhere in the Anglosphere, and 29.3% if the PCP was educated elsewhere. 65.6% of women who had changed addresses by one year after the index date reattended compared to 75.0% of those who had not.

Among those women screened at an OBSP facility, 14.1% failed to receive the outcome M between 11 months and 30 months after the re-exposure M, not later than December 31, 2010, compared to 43.3% of the other women. Failing to receive the outcome M was independently associated with a higher Charlson comorbidity score (OR = 1.06 per one unit increase in score, 95% confidence interval 1.03-1.09), having a male physician (OR = 1.05, 95% confidence interval 1.02-1.05), and having changed postal codes within one year after the re-exposure M (OR = 1.63, 95% confidence interval 1.53-1.73). Having the previous mammogram at an OBSP facility (OR = 0.18, 95% confidence interval 0.18-0.19), and having a Canadian trained (OR = 0.83, 95% confidence interval 0.80-0.87) or Anglosphere- trained physician (OR = 0.91, 95% confidence interval 0.84-0. 98) rather than a foreign-trained physician, were protective against failing to receive the outcome M (Table 2).

**Table 2:** **Factors affecting the likelihood of not reattending**

	Odds ratios for not reattending (95% CI)
Factor *	(N = 105,665)
Screening provider for baseline and re-exposure M	
OBSP	0.18 (0.18-0.19)
non-OBSP	Reference
Charlson comorbidity score	
Per point increase	1.06 (1.03-1.09)
Gender of woman’s physician	
Male	1.05 (1.02-1.05)
Female	Reference
Location of medical school of woman’s physician	
Canadian	0.83( 0.80-0.87)
Other Anglosphere	0.91 (0.84-0.98)
Elsewhere	Reference
Years in practice of woman’s physician, (per year)	1.0 (1.0-1.0)
Has a primary care physician one year after re-exposure M	
Yes	0.81 (0.68-0.97)
No	Reference
Postal code change 1 year after re-exposure M	
Yes	1.61 (1.52-1.71)
No	Reference

*This is a full model. All variables defined, described, and analyzed univariately and bivariately are included in this full model. All 2 way interactions were tested and none were significant.

## Discussion

Being screened at an OBSP facility is associated with a significantly lower likelihood of failing to receive subsequent breast screening, compared to being screened by PCP referral at non-OBSP facilities, after adjusting for Charlson comorbidity score, PCP gender, location of PCP training, residential mobility (using postal code change at one year), and whether the woman continued to have a usual PCP 12 months after the re-exposure M.

Others have reported that women with poorer self reported health are less likely to adhere to cancer screening [12], and specifically breast screening [13].

There is limited knowledge about the association of a woman’s residential mobility with her continuing adherence to screening mammography. Mobley et al. analyzed Medicare data on California women and showed that recent address change decreases the likelihood of using screening mammography services by 7% [14]. The current work shows that postal code change within a year of the index mammogram increases the likelihood of failure to receive the next periodic screening mammography. Residential mobility may result in failed delivery of the reminder, increased stress [13], or lack of a usual PCP to refer for screening.

Martin-Lopez et al. found that women who received a screening mammogram were more likely to have recently visited a PCP [15], and Esteva et al. found that participants in a public breast screening program visited their PCPs more frequently in the previous year than non participants [16]. Recommendations from PCPs increase breast screening participation [16,17], and it is likely that women who visit PCPs are exposed to more recommendations than those who do not. Duijm et al. found that barriers to receiving follow-up screening mammography were PCP-related rather than patient related in 70% of cases (in a study of women at risk for breast cancer due to a positive family history) [18]. Most of these barriers resulted from a failure of the PCP to recall that the patient was due for a screening mammogram. A programme such as the OBSP may avoid such barriers and may empower the patient by informing her directly when a mammogram is due.

It has been well described that female PCPs are more likely to encourage screening mammography and pap smears than their male counterparts [19-23]. In the current work we report a small association of the failure to receive the next periodic screening mammogram with male PCPs. Possible explanations may include a stronger orientation among female PCPs towards screening and preventive care, and believing more strongly in the effectiveness of mammography [23].

Women having a Canadian trained PCP (OR = 0.83, 95% confidence interval 0.80-0.87) or a PCP who trained in the Anglosphere (OR = 0.91, 95% confidence interval 0.84-0.98) were less likely to fail to receive the outcome M compared to those having a PCP trained elsewhere in the world (reference, OR = 1.0). Thind et al. analyzed the practice patterns of Ontario PCPs using self reported surveys and found that Canadian trained PCPs were more likely to provide appropriate screening and preventive care than international medical graduates [24]. Our findings corroborate this.

Sin and Leger conducted a systematic review of interventions designed to increase breast screening uptake, and identified 28 studies among 25 citations [25]. None of these studies specifically examined failure to receive the next periodic screening mammogram. Simple interventions such as reminders were found to be efficacious at improving attendance, and more complicated and costly interventions were not more efficacious. Our findings also show that simple reminder letters are associated with a large increase in the likelihood of reattendance.

We matched on age, income, and census tract as measured confounders, therefore estimates for those variables are not obtainable. There may be confounding factors not accounted for in the present analysis. Women who received the baseline M and re-exposure M at OBSP facilities may be more oriented towards screening and preventive health services than those who received the baseline M and re-exposure M at non-OHIP facilities upon referral by PCPs. These results are consistent with the hypothesis that reminder letters sent by the OBSP to women who are due to receive the next periodic screening mammograms prevent failure to receive the next screen, compared to the absence of such a reminder among women who receive screening on referral by the PCP to non-OBSP mammography facilities.

## Conclusion

Women in enrolled the Ontario Breast Screening Program (OBSP), a population-based breast screening programme sending reminder letters to women due for the next periodic screening, are less likely to fail to receive the next screening, compared to those who receive screening only when and if the PCP refers them to non-OBSP facilities. Women with higher comorbidity burden, those who change addresses after the index scan, and those without usual PCPs are more likely to fail to receive the next screen. The OBSP removes some of the barriers to breast screening and is effective at increasing mammography reattendance. Physician-referral based screening might achieve higher reattendance if routine invitations to reattend were to be adopted by the non-OBSP mammography facilities.

## References

[1] Jemal A, Bray F, Ferlay J (2011). Global cancer statistics. CA-A Cancer J Clin.

[2] Humphrey L, Helfand M (2002). Breast cancer screening: a summary of the evidence for the US Preventive Services Task Force. Ann Intern Med.

[3] Kerlikowske K, Grady D, Rubin S, Sandrock C, Ernster VL (1995). Efficacy of screening mammography: a meta analysis. JAMA.

[4] Elmore J, Armstrong K, Lehman C, Fletcher S (2005). Screening for breast cancer. JAMA.

[5] Force UPST (2009). Screening for breast cancer: US Preventive Services Task Force recommendation statement. Ann Intern Med.

[6] Fitzpatrick-Lewis D, Hodgson N, Ciliska D, Gorber SC, Bell N, Brauer P (2011). Breast cancer screening.

[7] Tatla R, Paszat L, Bondy S, Chen Z, Chiarelli A, Mai V (2003). Socioeconomic status & returning for a second screen in the Ontario breast screening program. Breast.

[8] Mayer J, Lewis E, Slymen D, Dullum J (2000). Patient reminder letters to promote annual mammograms: a randomized controlled trial. Prev Med (Baltim).

[9] Wagner T (1998). The effectiveness of mailed patient reminders on mammography screening: a meta-analysis. Am J Prev Med.

[10] Kiran T, Wilton AS, Moineddin R, Paszat L, Glazier RH (2014). Effect of payment incentives on cancer screening in Ontario primary care. Ann Fam Med.

[11] Lantz P, Stencil D (1995). Breast and cervical cancer screening in a low-income managed care sample: the efficacy of physician letters and phone calls. Am J Public Health.

[12] Yang T-C, Matthews SA, Hillemeier MM (2011). Effect of health care system distrust on breast and cervical cancer screening in Philadelphia, Pennsylvania. Am J Public Health.

[13] Lagerlund M, Drake I, Wirfält E, Sontrop JM, Zackrisson S (2015). Health-related lifestyle factors and mammography screening attendance in a Swedish cohort study. Eur J cancer Prev.

[14] Mobley LR, Kuo T-MM, Clayton LJ, Evans WD (2009). Mammography facilities are accessible, so why is utilization so low?. Cancer Causes Control.

[15] Martín-López R, Jiménez-García R, Lopez-de-Andres A, Hernández-Barrera V, Jiménez-Trujillo I, Gil-de-Miguel A (2013). Inequalities in uptake of breast cancer screening in Spain: analysis of a cross-sectional national survey. Public Health.

[16] Esteva M, Ripoll J, Leiva A, Sánchez-Contador C, Collado F (2008). Determinants of non attendance to mammography program in a region with high voluntary health insurance coverage. BMC Public Health.

[17] Phillips KA, Kerlikowske K, Baker LC, Chang SW, Brown ML (1998). Factors associated with women’s adherence to mammography screening guidelines. Health Serv Res.

[18] Duijm L, Guit G, Zaat J (1997). Mammographic surveillance of asymptomatic breast cancer relatives in general practice: rate of re-attendance and GP-and patient-related barriers. Fam Pract.

[19] Lurie N, Slater J, McGovern P, Ekstrum J, Quam L, Margolis K (1993). Preventive care for women–does the sex of the physician matter?. NEJM.

[20] Haggerty J, Tamblyn R, Abrahamowicz M, Beaulieu MD, Kishchuk N (1999). Screening mammography referral rates for women ages 50 to 69 years by recently-licensed family physicians: physician and practice environment correlates. Prev Med (Baltim).

[21] Franks P, Clancy C (1993). Physician gender bias in clinical decisionmaking: screening for cancer in primary care. Med Care.

[22] Bulliard J-L, De Landtsheer J-P, Levi F (2004). Reattendance in the Swiss mammography screening pilot programme. J Med Screen.

[23] Lurie N, Margolis KL, McGovern PG, Mink PJ, Slater JS (1997). Why do patients of female physicians have higher rates of breast and cervical cancer screening?. J Gen Intern Med.

[24] Thind A, Feightner J, Moira S, Cathy T, Andrea B (2008). Who delivers preventive care as recommended? Analysis of physician and practice characteristics. Can Fam Physician.

[25] Sin JP, St Leger AS (1999). Interventions to increase breast screening uptake: do they make any difference?. J Med Screen.

